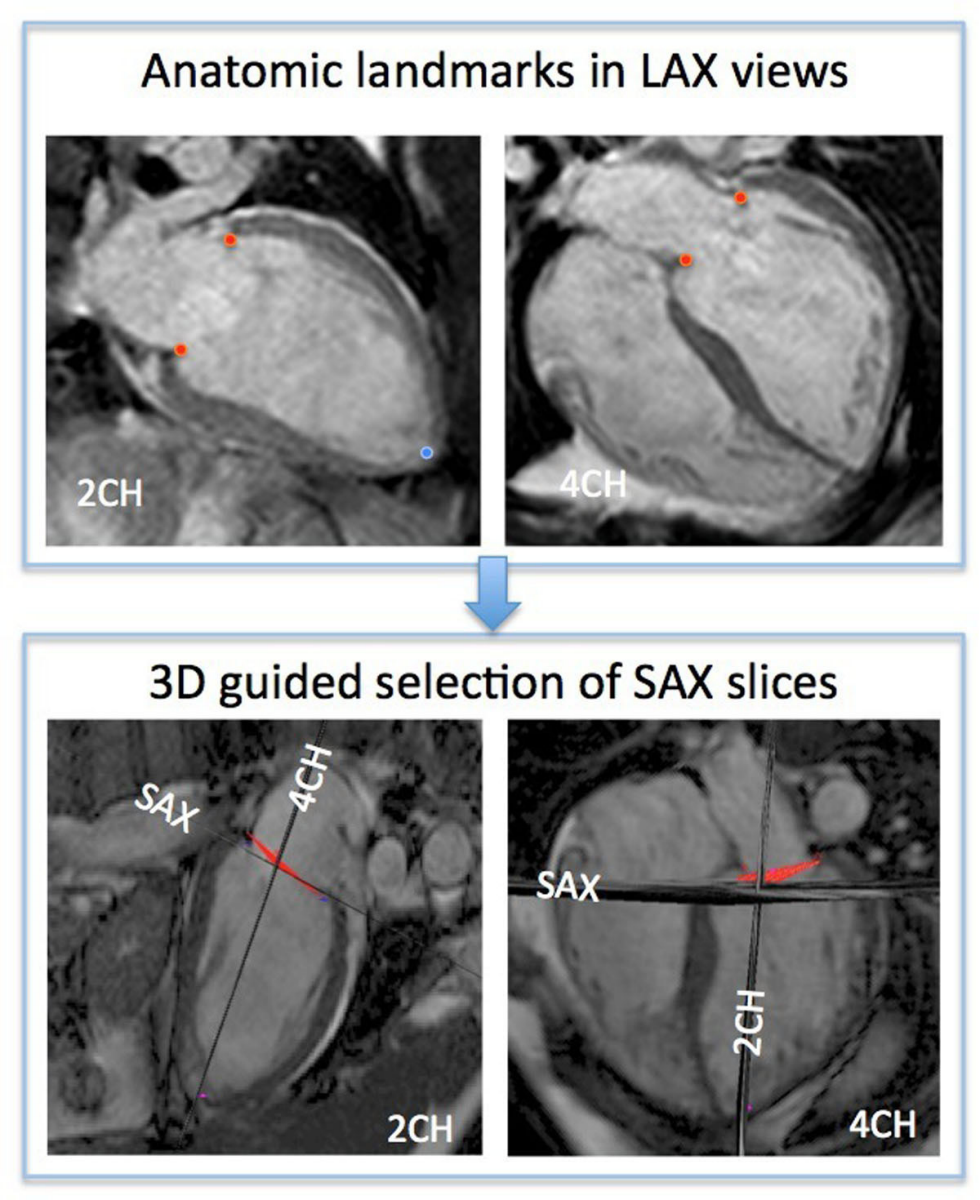# Objective selection of short-axis slices for automated quantification of left ventricular size and function by cardiovascular magnetic resonance

**DOI:** 10.1186/1532-429X-17-S1-P392

**Published:** 2015-02-03

**Authors:** Marco Marino, Victor Mor-Avi, Francesco Maffessanti, Cristiana Corsi, Amit R Patel

**Affiliations:** 1 University of Chicago, Chicago, IL, USA; 2 University of Bologna, Bologna, Italy

## Background

Quantification of left ventricular (LV) size and function from CMR images relies on endocardial border delineation and selection of relevant short-axis (SAX) slices. Multiple border detection algorithms have been tested to replace subjective manual tracing. However, there are no widely accepted criteria which basal slices to include in the LV volume, and endocardial definition near the apex is often suboptimal, making this task challenging in many patients. We hypothesized that this could be solved by defining mitral annular (MA) plane and apex in the long-axis (LAX) views. Our goal was to test this approach in combination with automated endocardial border detection using conventional methodology as a reference.

## Methods

SAX images from 50 patients were analyzed using custom software to measure end-systolic and end-diastolic LV volumes (ESV, EDV) and ejection fraction (EF) from stacks of SAX images. The apex and insertion points of the mitral leaflets were manually marked on LAX views, and used to approximate MA plane at ED and ES. Automated measurement of LV volumes was guided by including in the LV cavity only slices or parts of slices located between MA plane and the LV apex. Endocardial border was automatically detected using our previously described algorithm based on a probabilistic level set approach guided by noise distribution. Endocardial boundaries were also manually traced by an experienced investigator to obtain reference values. To determine the main sources of inter-technique disagreement, 10 patients with largest errors were reviewed using combined 3D displays of the LAX and relevant SAX views with the MA plane.

## Results

Selection of the anatomic landmarks in LAX views was possible in all 50 patients, allowing automated measurement of LV volumes without the need for subjective slice selection. Inter-technique comparisons resulted in high correlations (EDV: r=0.95; ESV: r=0.96; EF: r=0.77), small biases (1 and 9 ml and -7%) and reasonable limits of agreement. 3D visualization showed that the worst inter-technique agreement was due to incorrect manual tracing at LV base that erroneously included parts of the atrium or excluded parts of LV cavity in 7/10 patients, and other reasons in the remaining 3 patients.

## Conclusions

Defining the MA plane and apex in the LAX views obviates the need for subjective decision on slice inclusion and LV cavity boundary delimitation in basal slices. When combined with automated endocardial boundary detection, it provides measurements of LV volumes and function that are potentially more accurate than the standard methodology.

## Funding

None.

**Figure 1:**